# Editorial: Bioactive compounds in neuroprotection: unveiling modern therapeutics in aging

**DOI:** 10.3389/fnagi.2026.1956060

**Published:** 2026-08-18

**Authors:** Chandra Prakash, Pavan Kumar, Deepak Sharma

**Affiliations:** 1 Neurobiology Laboratory, School of Life Sciences, Jawaharlal Nehru University, New Delhi, India; 2 Department of Anatomy and Cell Biology, College of Medicine, The University of Illinois at Chicago, Chicago, IL, United States

**Keywords:** aging, bioactive compounds, neurodegenerative disorders, neuroprotection, therapeutics

Aging is a progressive, natural decline in biological functions that is often associated with neurodegenerative diseases (NDDs) such as Alzheimer's disease (AD) and Parkinson's disease (PD). Distinct pathological features, including structural and functional impairments of the nervous system, are the hallmark of these diseases (Tesco and Lomoio, 2022; Gadhave et al., 2024). As they predominantly affect the elderly population worldwide (Zhou et al., 2024), they have become a major focus of neuroscience research. Numerous therapeutic strategies have been developed to treat NDDs (Sepehrimanesh et al., 2026). However, the precise mechanisms underlying many of these strategies remain incompletely understood, limiting their therapeutic efficacy. Bioactive compounds are naturally occurring chemical substances derived from plants, animals, and microorganisms that possess a wide range of biological properties (Ali et al., 2026). These compounds exert neuroprotection by modulating cellular signaling pathways, reducing oxidative stress, and suppressing neuroinflammation, thereby preserving neuronal functions and integrity (Babazadeh et al., 2023). Consequently, they hold significant potential to mitigate neurodegeneration in aging and help preserve cognitive and motor functions.

This Research Topic comprises five articles highlighting recent therapeutic advances in bioactive compounds for the management of NDDs. AD is one of the most prevalent and complex NDD, characterized by progressive cognitive decline. It is also a leading cause of disability and morbidity among the aging population (Safiri et al., 2024). Ambroxol hydrochloride is a synthetic derivative of the natural alkaloid vasicine isolated from *Justicia adhatoda*, that possesses antioxidant and anti-inflammatory properties (Gupta, 2010). Ahmad et al. investigated the therapeutic efficacy of ambroxol in a scopolamine-induced mouse model of AD. The study demonstrated that ambroxol attenuated scopolamine-induced oxidative stress, neuroinflammation, and neurodegeneration. Moreover, the bioactive compound reduced lipid peroxidation and reactive oxygen species while enhancing the expression of key antioxidant defense enzymes. It also suppressed c-Jun N-terminal kinase activation, restored dysregulated Akt and glycogen synthase kinase-3β signaling, and attenuated reactive gliosis and inflammation. Furthermore, ambroxol preserved synaptic integrity and significantly improved cognitive function, highlighting its potential as a promising therapeutic candidate for AD.

Citrus fruits are rich in bioactive compounds and exhibit biological activities, including antioxidant (Borghi and Pavanelli, 2023) and anti-inflammatory (Fontana et al., 2022) properties. Liu et al. investigated the therapeutic potential of citrus plants using an integrated framework combining network pharmacology, molecular docking, transcriptomic validation, and experimental verification. The comprehensive analysis identified multiple flavonoids, including quercetin, nobiletin, hesperidin, hesperetin, apigenin, and tangeretin, as key multitarget agents capable of simultaneously regulating neuroinflammation, oxidative stress, acetylcholine metabolism, apoptosis, and ferroptosis. Finally, experimental validation demonstrated that hesperidin and naringin attenuated microglial cell activation, suggesting the potential of these bioactive compounds in AD.

The research paper by Tavares et al. demonstrated the therapeutic potential of bioactive peptides isolated from the green macroalga, *Ulva rigida*. The authors optimized peptide extraction and identified antioxidant fractions that inhibit β-amyloid aggregation. The article also addressed poor bioavailability and blood-brain-barrier (BBB) permeability by encapsulating peptides in solid lipid nanoparticles. The nanoformulation improved peptide stability and provided an efficient delivery platform for brain-targeted therapy. Overall, the work exemplifies how marine-derived bioactive compounds, when combined with advanced nanocarrier systems, may overcome longstanding translational limitations in NDD treatment.

PD is another common NDD characterized by loss of motor function, including resting tremor, muscular rigidity, bradykinesia, and postural instability (Chen et al., 2022). Although the precise pathogenic mechanisms underlying PD remain incompletely understood, growing evidence indicates that neuroinflammation and immune dysregulation play pivotal roles in the initiation and progression of the disease (Lind-Holm Mogensen et al., 2025). Congrong Shujing Granules (CRSJ), a traditional Chinese medicine formulation, is known to alleviate PD symptoms (Chen et al., 2020). Li et al. reported that CRSJ treatment significantly improved motor function, preserved dopaminergic neurons, and reduced α-synuclein accumulation in the PD mouse model by restoring Th17/Treg immune homeostasis, suppressing the CX3CL1/CX3CR1 and TGF-β/SMAD3 pathways, and reducing microglial activation and neuroinflammation. Overall, the paper provides compelling mechanistic insight supporting the development of CRSJ as a potential immunoregulatory therapeutic strategy for PD.

Gerobiotics are an emerging class of probiotics, and their derived postbiotics have the potential to target aging, modulate the gut-brain axis, and alleviate age-related cognitive decline. Gerobiotics can also increase the production of a variety of bioactive compounds, which can mitigate systemic dysfunctions and aging (Ma et al., 2023). The review by Kocaadam-Bozkurt et al. discussed the neuroprotective effects of gerobiotics. Both preclinical and clinical studies have reported that specific gerobiotics can modulate the microbiota-gut-brain axis, reduce neuroinflammation, enhance synaptic function, balance immune responses, and restore BBB integrity, ultimately improving cognitive performance. Furthermore, the review advocates for the development of microbiome-based therapeutic strategies that can increase the levels of bioactive compounds and enhance therapeutic efficacy, thereby reducing the required dosage of conventional pharmacological treatments.

In summary, the articles published in this Research Topic present intriguing results that advance understanding of the age-related progression of NDDs and their treatment. However, these treatments often have some side effects. Thus, there is an urgent need to further investigate the effectiveness and mechanisms of action of traditional medicinal herbs and their bioactive compounds to develop safe and effective treatments for NDDs. We believe that this Research Topic will significantly expand our current understanding of this field and help us develop therapies based on bioactive compounds.
